# Investigating the Precise Localization of the Grasping Action in the Mid-Cingulate Cortex and Future Directions

**DOI:** 10.3389/fnhum.2022.815749

**Published:** 2022-02-24

**Authors:** Zebunnessa Rahman, Nicholas W. G. Murray, Jacint Sala-Padró, Melissa Bartley, Mark Dexter, Victor S. C. Fung, Neil Mahant, Andrew Fabian Bleasel, Chong H. Wong

**Affiliations:** ^1^Department of Neurology, Westmead Hospital, Sydney, NSW, Australia; ^2^Westmead Clinical School, University of Sydney, Sydney, NSW, Australia

**Keywords:** grasping action, cingulate cortex, cingulate motor area, electrical stimulation, stereo EEG

## Abstract

**Objective:**

To prospectively study the cingulate cortex for the localization and role of the grasping action in humans during electrical stimulation of depth electrodes.

**Methods:**

All the patients (*n* = 23) with intractable focal epilepsy and a depth electrode stereotactically placed in the cingulate cortex, as part of their pre-surgical epilepsy evaluation from 2015 to 2017, were included. Cortical stimulation was performed and examined for grasping actions. Post-implantation volumetric T1 MRIs were co-registered to determine the exact electrode position.

**Results:**

Five patients (male: female 4:1; median age 31) exhibited contralateral grasping actions during electrical stimulation. All patients had electrodes implanted in the ventral bank of the right cingulate sulcus adjacent to the vertical anterior commissure (VAC) line. Stimulation of other electrodes in adjacent regions did not elicit grasping.

**Conclusion:**

Grasping action elicited from a localized region in the mid-cingulate cortex (MCC) directly supports the concept of the cingulate cortex being crucially involved in the grasping network. This opens an opportunity to explore this region with deep brain stimulation as a motor neuromodulation target for treatment in specific movement disorders or neurorehabilitation.

## Introduction

The hand grasping mechanism is a motor phenomenon of great interest to clinicians and researchers for its value in the localization of cortical damage (Fulton, 1934; Seyffarth and Denny-Brown, 1948) and the epileptogenic zone (Horinouchi et al., 2018).

Historically, lesional studies have primarily localized the grasping mechanism to the frontal lobe, including the supplementary motor area (SMA) and cingulate cortices (Fulton, 1934; Seyffarth and Denny-Brown, 1948; Hashimoto and Tanaka, 1998). A few studies have reported grasping by direct cortical stimulation (Talairach et al., 1973), particularly when targeting the mid-cingulate cortex (MCC) (Talairach et al., 1973; Chassagnon et al., 2008; Caruana et al., 2018).

The cingulate cortex is anatomically diverse with extensive connectivity to other cortical and subcortical regions involved in planning and complex movement (Chassagnon et al., 2008). Based on cytoarchitectonics, immunohistochemistry, and myelinization studies, the cingulate cortex in humans is divided into four regions: anterior cingulate cortex (ACC), mid cingulate cortex (MCC), posterior cingulate cortex (PCC), and retrosplenial cortex (RSC). These regions are further divided into eight subregions; with the ACC divided into subgenual (sACC) and pregenual ACC (pACC), MCC into anterior and posterior MCC (aMCC, pMCC), PCC into ventral and dorsal PCC (vPCC, dPCC), and RSC into dorsal and ventral RSC (dRSC, vRSC) (O’Neill et al., 2009).

**FIGURE 1:**
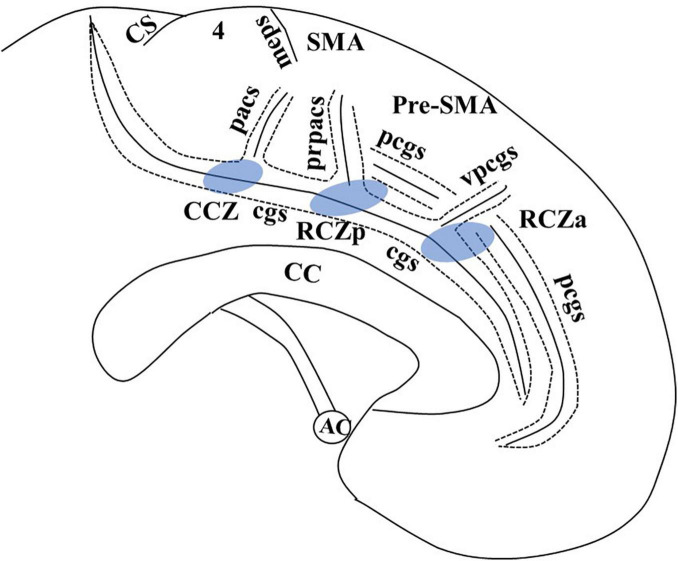
Adapted from Amiez and Petrides (2014), with email permission. CC, corpus callosum; CCZ, caudal cingulate zone (marked in blue); cgs, cingulate sulcus; CS, central sulcus; meps, medial paracentral sulcus; pcgs, paracingulate sulcus; pacs, paracentral sulcus; pr-pacs, pre-paracentral sulcus; RCZa, rostral cingulate zone anterior (marked in blue); RCZp, rostral cingulate zone posterior (marked in blue); SMA, supplementary motor area; vpcgs, vertical paracingulate sulcus. We have taken email permission for reproducing Figure 1 from Céline Amiez (celine.amiez@inserm.fr).

The MCC is unique from other cingulate regions due to the presence of motor areas buried within the cingulate sulcus (cgs). This region, also known as the cingulate motor area (CMA), has generated great interest among researchers. In macaque monkeys, intracortical microstimulation and cortical fiber tracking by tracer injection suggest three distinct CMAs buried within the cgs, each of which corresponds to a different cytoarchitectonic field (Dum and Strick, 1991; Luppino et al., 1991; Shima et al., 1991). In humans, the identification of primitive gigantopyramidal neurons in the cingulate sulcus in autopsy studies first suggested the presence of a motor field in the MCC (Braak, 1976; Braak and Braak, 1976). Later studies incorporating functional PET (Picard and Strick, 1996, 2001) and functional magnetic resonance imaging (MRI) (Amiez and Petrides, 2014) proposed a comparable organization of CMA in humans like those in non-human primates. These are defined as anterior rostral cingulate zone (RCZa), posterior rostral cingulate zone (RCZp), and caudal cingulate zone (CCZ) (Figure 1). The cingulate sulcus making the superior boundary of the cingulate cortex in humans is more complex than primates due to multiple segmentation, with considerable intersubject variability (Amiez and Petrides, 2014). In addition, there may be a second sulcus running parallel and dorsal to the cgs in the medial surface known as the paracingulate sulcus (pcgs) (Vogt et al., 1995, 2005; Amiez et al., 2018). Although the exact localization has not been clearly defined, Picard and Strick (1996) in their functional PET review suggested the localization of RCZp is 1 mm anterior to the vertical anterior commissure (VAC) line buried within the cgs, RCZa 24 ± 7 mm anterior to VAC line and CCZ posterior to the VAC line (Figure 1). One limitation of Picard and Strick’s (1996) work is that localization was based on the CT brain (Amiez and Petrides, 2014). Further study with fMRI by Amiez and Petrides (2014) indicated the localization of CCZ below the paracentral lobule, RCZp at the level of SMA, and RCZa further anterior to RCZp within the cingulate/paracingulate sulcus. They also demonstrated that the CMAs are somatotopically organized, and eye and tongue/mouth representation is only appreciated in RCZa and RCZp.

Direct cortical stimulation is a powerful scientific tool that allows the opportunity to map certain functions of the cerebral cortex by reproducing signs or movements (such as hand grasping) with targeted stimulation (Penfield and Boldrey, 1937; Lesser et al., 1987; Selimbeyoglu and Parvizi, 2010). Previous studies have generated grasping using cortical stimulation in epilepsy patients (Talairach et al., 1973), particularly when targeting the MCC (Talairach et al., 1973; Chassagnon et al., 2008; Caruana et al., 2018). These studies, however, have primarily observed the grasping mechanism in single cases or through retrospective review, meaning they have not incorporated systematic and well-defined examination protocols. Implementing a predefined examination protocol in a prospective sample allows researchers to systematically investigate which regions reliably produce the grasping mechanism following stimulation and the influence of certain stimulation parameters. This could ultimately inform researchers exploring the manipulation of hand movements using stimulation in other clinical cohorts.

Creating an artificial grasping system may have serious implications beyond epilepsy, for example, when attempting to restore the function of the hand in patients with complete paralysis of the limb following a stroke (Schwartz et al., 2009). However, studies exploring such options are sparse. Further knowledge of the accurate localization and mechanism of grasping in the cingulate cortex may be valuable to explore this possibility in the near future.

Here we prospectively explored the nature of the grasping mechanism using electrical stimulation in patients who had intracranial electrodes placed for pre-surgical evaluation of their medically intractable epilepsy. Specifically, we aimed to localise the grasping action within the MCC in a T1 volumetric MRI, and explore whether it is a purely provoked phenomenon or if sensory input is required during electrical stimulation, using a test protocol modified from previous literature (Table 1).

**TABLE 1:** Methods of eliciting varieties of grasping actions and the expected responses (modified from Hashimoto and Tanaka, 1998).

	Stimulus	Response
**Grasping actions**		
Grasp reaction	Tactile stimuli applied to the palm	Flexion and adduction of the fingers and thumb to grab examiner’s finger
**Instinctive reaction**		
Closing reaction-Method 1	Moving tactile stimuli of the examiner’s finger at the back of the hand between thumb and index fingers	Turn hand and grab examiner’s finger
Closing reaction-Method 2	A light stationary or moving touch in a circle completed by thumb and index finger	A sequence of closing movements of the hand that brings the stimulus (examiner’s finger) to the center of the palm
Trap reaction	Moving tactile stimuli of the examiner’s fingers away from the patient’s palm	A sudden tightening or flexion of fingers to grasp the examiner’s fingers
Magnet reaction	A retreating light touch by the examiner’s finger on the patient’s fingertips	Pursuing movements of the arm and hand to keep in contact with the stimulus to finally grab it
Visual reaction	The visual presentation of the examiner’s finger or a pen	Pursuing movements of the arm and hand to the stimulus to grasp

## Materials and Methods

Patients were prospectively recruited from the Epilepsy Monitoring Unit at Westmead Hospital, while undergoing Stereotactic electroencephalogram (EEG) monitoring (SEEG) from March 2015 to April 2017. Patients underwent SEEG because non-invasive investigations could not accurately define the epileptogenic cortex. SEEG involves the surgical implantation of depth electrodes, inserted stereotactically through 2-mm burr holes at multiple locations, which measure brain activity from lateral, basal, and mesial surfaces of the cerebral cortex. Since each electrode has multiple contacts, this produced a 3D recording matrix from different cerebral regions. Patients were included in the following study if they had at least one depth electrode implanted in the cingulate cortex. Implantation of cingulate cortex electrodes was based on the patient’s pre-surgical evaluation, involving careful review of all previous non-invasive investigations (seizure semiology, VEEG, MRI, SPECT, PET, and neuropsychological assessment) (McGonigal et al., 2007).

### Implantation and Recording

DIXI electrodes (DIXI Medical, France, United Kingdom) were used for SEEG implantation. Each electrode was 0.8 mm in diameter and varied between 8 and 18 contacts depending on the site of implantation. Each contact was 2 mm in length separated by a 1.5 mm plastic insulator. The surface area of each electrode directly in contact with brain tissue was 0.05 cm^2^ (Medtronic, 2010a,b) for SEEG recording and cortical stimulation. Following SEEG implantation, continuous intracerebral recordings of all electrodes commenced day 2 of the SEEG electrode implantation. The duration of the recordings varied from 8 to 10 days depending on the information obtained. Electrical stimulation was usually performed on days 7–9 post-implantation. Patients were returned to their usual antiepileptic medications at least 12 h before stimulation, as per the normal SEEG stimulation protocol (Ritaccio et al., 2018).

### Stimulation and Analysis of Grasping Mechanism

A protocol for systematically screening and eliciting the grasping mechanism was established for all prospective patients following the observation of grasping in our index case. This was modified from a previously published method for eliciting grasping actions (Table 1; Hashimoto and Tanaka, 1998). We screened for grasping by using biphasic current with 20 Hz stimulation, with a stepwise increase in the intensity of 3, 6, 9, and 12 mA. If grasping actions did not occur, stimulation frequency was increased to 50 Hz (stepwise current intensity: 3, 6, and 9 mA; pulse width: 0.3 ms, and duration of current: 5 s). We did deviate from this protocol in case 2 applying 10 Hz current and in case 3 applying up to 8 s duration of the current. Each examination maneuver detailed in Table 1 was performed a single time at each level of electrical stimulation during screening.

Patients who showed grasping actions on screening, either with 20 or 50 Hz stimulation, were further selected for a more detailed examination protocol (Table 1) using the same current parameter at 10, 20, and 50 Hz, with stepwise increasing current intensity. Bipolar stimulation was performed using two adjacent contacts. The electrodes eliciting grasping actions were then stimulated with an inactive referential electrode located in the subcortical white matter in the frontal or parietal lobe to determine the electrode with the lowest current provoking the grasping actions for localization. Electrical stimulation was performed over 4–5 sessions, lasting 1–2 h each. Each examination maneuver detailed in Table 1 was examined 5–6 times at each level of electrical stimulation. These were examined over separate sessions to confirm the presence or absence of various actions and the latency from stimulus to onset was documented. We examined the hand contralateral and ipsilateral to the side of electrical stimulation before, during, and after the electrical stimulation, using the protocol. The electrical stimulation was performed twice with sensory stimuli (tactile and visual), once without any sensory stimulus, once with an ipsilateral hand, and then repeated if clarification was required. After establishing the lowest current that reliably evoked various grasping actions, patients were instructed not to grab the target (finger or a pen of examiner) during stimulation to determine whether grasping actions could be suppressed by the patient. The EEG was recorded continuously during stimulation to control for after-discharges or EEG seizures.

The charge density delivered at each electrode was calculated in μC (microcoulomb) per cm^2^ per pulse. The calculated charge density for 3, 6, 9, and 12 mA was 18, 36, 54, and 72 μC/cm^2^ per pulse, respectively.

Mean and the standard error of the mean were used for the duration and the latency of grasping action. Spearman’s correlation was applied to detect the correlation of the current intensity with the latency and duration of grasping. Probability value (*p*) < 0.05 was considered significant. SPSS (IBM Corp. Released 2016. IBM SPSS Statistics for Windows, Version 24.0. Armonk, NY: IBM Corp.) was used for the statistical analysis.

### Functional Mapping and Localization of the Stimulation Site

In patients with positive grasping actions, the localization of the electrode of interest was displayed concerning the VAC line in the original sagittal T1 MRI. The anterior commissure-posterior commissure line (AC-PC) passes between the superior edge of the anterior commissure and the inferior edge of the posterior commissure. VAC line is the vertical line at the posterior edge of the AC perpendicular to the AC-PC line (Talairach et al., 1973).

To determine the position of the electrode contacts in the cingulate cortex, post-SEEG T1 volumetric MRI of each subject was normalized to the ICBM152 brain template using the Statistical Parametric Mapping (SPM) software version 12. The cingulum flat map and co-registration method described by O’Neill et al. (2009) was then applied to define the four regions and eight subregions of the cingulate cortex as per O’Neill et al. (2009).

## Results

**FIGURE 2:**
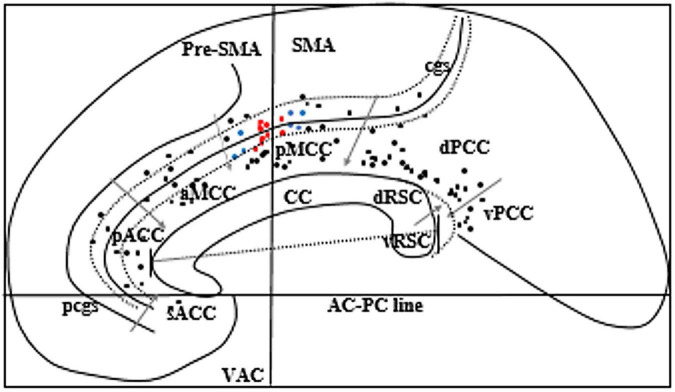
Schematic sagittal view of the ICBM152 brain 5 mm right to the midline. Corpus callosum (CC) was rotated to superimpose with a “Flat map” of O’Neill et al. (2009) to locate eight subregions of the cingulate gyrus. We rotated the flat map to show the vertical alignment of the VAC line. Gray arrows indicate the margins of each subregion of the cingulate gyrus. Black dots represent all cingulate electrodes. Red dots represent the electrodes producing grasping actions, and blue dots represent the electrodes producing various motor phenomena other than grasping actions. Two long-curved dotted lines on each side of the cingulate sulcus (cgs) represent the ventral and the dorsal bank of cgs. The straight line across the CC indicates the anteroposterior dimension of the CC. The dotted curved line posterior to the splenium of CC represents the callosal sulcus to show the location of RSC in the depth of the callosal sulcus. pcgs, Paracingulate sulcus. Two electrode contacts (red dots) are overlapping.

A total of 23 patients (male = 13, female = 10) had at least one electrode implanted in the cingulate cortex during the study period. The median age during SEEG was 25 years. Ten had right-sided implantation, eleven had left-sided implantation, and two had bilateral implantation. Nine patients had a paracingulate sulcus (pcgs) parallel to the cingulate sulcus on the implanted side. The anatomy of the sulcus was variable including continuous or segmented cingulate sulcus and continuous or segmented pcgs. A total of 105 cingulate contacts were identified, 2 in sACC, 12 in pACC, 13 in aMCC, 15 in pMCC, 12 in the cingulate sulcus anterior to VAC line within pMCC, 13 in the cingulate sulcus posterior to VAC line within pMCC, 32 in dPCC, and 6 in vPCC (Figure 2).

Five patients (male = 4, all right-handed) showed grasping actions in the hand contralateral to electrical stimulation of the cingulate cortex. Grasping actions were localized to a region in pMCC anterior to the VAC (detailed below, and Figure 2). The median seizure onset age for these patients was 8 years. The average duration of epilepsy was 20 years (13–39 years). One patient (case 3) had mild left-hand apraxia; the other four patients had a normal neurological examination. None of them showed grasping actions without electrical stimulation. All patients had right-sided implantation as their non-invasive epilepsy investigations lateralized the seizure onset to the right hemisphere. SEEG confirmed that none of these five patients had seizure onset from the cingulate gyrus. No after-discharges or EEG seizures were recorded during stimulation of the cingulate cortex. Details of each patient’s seizure characteristics, EEG, SEEG, MRI, PET, and neuropsychological findings are described in Table 2.

**TABLE 2:** Demographics, seizure characteristics, EEG, SEEG, neuroimaging findings, neuropsychological assessment, surgical outcome, and histopathology of the epileptogenic cortex of the patient.

	Case 1	Case 2	Case 3	Case 4	Case 5
**Age (year)/Gender**	39/F	30/M	39/M	22/M	25/M

**Age at Sz onset**	26 years	8 years	3 weeks	18 months	14 years

**Neurological examination**	Normal	Normal	L hand apraxia	Normal	Normal

**Seizure semiology**	Sensory aura progressing to tonic posturing of left upper limb	Sensory aura progressing to tonic posturing of left upper and lower limb	1. Ictal pouting progressing to peri-oral clonic seizure followed by right manual automatisms 2. Ictal pouting progressing to bilateral asymmetric tonic posturing of upper limbs	Automatisms followed by left head turn progressing to bilateral tonic clonic seizure	Ictal pouting followed by left head turn associated with tonic posturing of left upper limb with progression to bilateral tonic clonic seizure

**Interictal EEG (Scalp) **	Bi-frontal	Nil	1. R frontal 2. Bi frontal	1. R Frontal, 2. Bifrontal, 3. R temporal	R fronto-temporal

**Ictal EEG** **(Scalp)**	Regional R fronto-central	Bilateral	Bilateral	1. R frontal 2. Bi-frontal 3. Non-localisable	R fronto-temporal

**SEEG** **Sz onset**	R Anterior insula	R posterior insula	1. R mesial frontal 2. R mesial temporal	R mesial fronto-polar/Anterior insular margin	Non-localising

**MRI**	Normal	Normal	1. Atrophic whole R hemisphere 2. FLAIR hyperintensity right insula, temporal-parietal region, 3. R mesial temporal sclerosis	R frontal lobe: thickened cortex, blurred gray white margin	2 mm FLAIR hyperintensity at the R frontopolar region (subcortical/white matter)

**FDG-PET**	Diffuse R fronto-parietal hypometabolism	Normal	1. Moderate hypometabolism of R hemisphere (except occipital lobe) 2. Severe hypometabolism R perisylvian temporoparietal lobe	Diffuse R frontal hypometabolism	Bilateral mild antero-mesial temporal hypometabolism

**Neuropsychology**	Normal memory. Below Average verbal intellectual abilities	Below average verbal intellectual abilities. Weakness in immediate attention span, working memory and speed of information processing	Mild to moderately impaired verbal intellect, extremely low non-verbal intellect. Normal language function. Significantly reduced attention.	Average verbal memory. Normal intellectual abilities. Slowing of information processing. Impaired attention.	Below average verbal and non-verbal intellectual skills, working memory, processing speed, verbal fluency. Low to low average both verbal and non-verbal memory.

**Surgical outcome**	Not performed (patient left the country)	Engel 1	Engel 1	Engel IV	Not performed

**Histology**	Not applicable	Diffuse neuronal heterotopia and type 1A cortical dysplasia	Neuronal loss, marked gliosis, dystrophic calcification, and corpora amylacea	MOGHE (mild malformation of cortical dysplasia with oligodendroglial hyperplasia and epilepsy)	Not applicable

*Bi, Bilateral; R, right; L, left; Sz, seizure.*

### Analysis of Electrically Induced Clinical Responses

The characteristics of the grasping actions elicited during stimulation of the electrodes with the lowest frequency and intensity of current in an individual patient are described in Table 3. Grasping actions were only produced from the hand contralateral to electrical stimulation. To exclude false-positive results, we examined for grasping as per the protocol while stimulating electrodes outside the cingulate region or without any electrical stimulation. Case 1 is our index patient who did not have all of the maneuvers examined, as the protocol was established after her evaluation.

**TABLE 3:** Grasping actions elicited with the lowest electrical stimulation parameter for individual patients.

Grasping actions									
**Grasping produced with afferent stimulation**									**Evoked G**

**Case No**	**Electrode**	**Hz**	**GR**	**CR1**	**CR2**	**TR**	**MR**	**VR**	

			**mA**	**mA**	**mA**	**mA**	**mA**	**mA**	
1	E4–E3	20	P	NE	NE	P	NE	P	P
			8 mA			8 mA		8 mA	8 mA
2	L2–L3	10	P	P	P	P	P	P	A
			3 mA	3 mA	3 mA	3 mA	3 mA	6 mA	
3	H3–H4	20	P	P	P	P	P	P/Ab	A
			6 mA	6 mA	9 mA	9 mA	9 mA	12 mA	
4	G2–G1	20	P	P	P	P	P	P/Ab	A
			6 mA	9 mA	9 mA	9 mA	9 mA	12 mA	
5	G3–G4	20	P	P	P	P	P	P/Ab	P
			3 mA	3 mA	9 mA	6 mA	6 mA	3 mA	3 mA

*A, absent at 50 Hz 10 mA current; CR1, closing reaction method 1; CR2, closing reaction method 2; GR, grasp reaction; Hz, frequency of electrical stimulation; MR, magnet reaction; mA, lowest current required to produce a response; NE, not examined; P, present; A, absent; P/Ab, present but could abort with prompting; evoked G, grasping evoked without tactile or visual stimulation; TR, trap reaction; VR, visual reaction.*

Although cases 2, 3, and 4 showed grasping actions only after applying the cutaneous or visual stimuli as per Table 1, grasping in cases 1 and 5 were evoked during electrical stimulation without applying any sensory input. They spontaneously approached the objects in the peripersonal space, such as their bedsheet or own clothes, to explore and grasp the objects. These were provoked irrespective of whether the palm was facing up or touching the bed sheet or their own clothes. In cases 1 and 5, grasping was also seen with arms outstretched, palm and dorsum of the hand not touching any objects, irrespective of the eyes being opened or closed. All four patients (cases 2, 3, 4, and 5) who were examined according to the protocol described in Table 1 consistently showed all forms of grasping actions on electrical stimulation. We found the final response with all of the maneuvers was grasping the examiner’s finger/pen firmly and not letting it go.

All the types of grasping actions except the visual reaction were elicited with the charge density between 18 and 54 μC/cm^2^/phase at 10 or 20 Hz. Visual reactions in cases 3 and 4 were produced with a charge density of 72 μC/cm^2^/phase at 20 Hz frequency.

**FIGURE 3:**
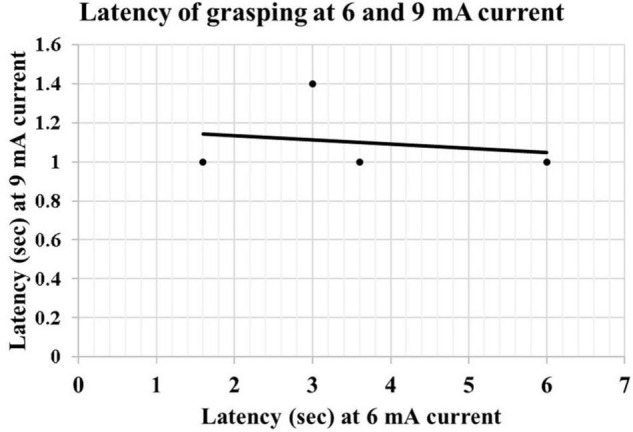
Scatter graph showing the correlation of latency of grasping at 6 and 9 mA current.

We observed that at low currents, grasping actions stop with the cessation of the electrical stimulation; however, at higher currents, the duration of the grasping exceeded the duration of electrical stimulation. After discharges were not seen in the EEG. The increase in the mean duration of the grasping reaction between 6 mA (0.15 ± 0.15 s) and 9 mA (1.54 ± 0.72 s) was statistically significant (*p* < 0.01, *R* = 0.84). There was a negative correlation between the intensity of the current and latency of onset of grasping reaction from the application of electrical stimulation at 6 mA (2.95 ± 1.19 s) and 9 mA (1.08 ± 0.08 s). However, this did not reach statistical significance (*P* = 0.051, *R* = −0.664) (Figure 3). The first case *did not have electrical stimulation at 6 mA current; therefore, excluded from the* statistical analysis.

### Voluntary Suppression of Visual Reaction

Cases 3–5 could abort the visual reaction after repeated prompting not to hold on to the examiner’s finger/pen placed in the visual field even at 72 μC/cm^2^/phase. Even though these patients managed to abort the visual reaction during electrical stimulation, they reported a persistent urge to grab the target, and irregular finger and wrist movements were noted during electrical stimulation. On asking the patients about the finger movement, they stated that their “hand wanted to move”; however, they managed to resist it. We did not find any difference in the characteristics of visual reaction on placing the finger/pen of the examiner in different visual fields within or outside of an arm’s length. When the target was placed out of arm’s length, patient would sit up to approach the target.

### Localization of Cortical Area Inducing Grasping

**FIGURE 4:**
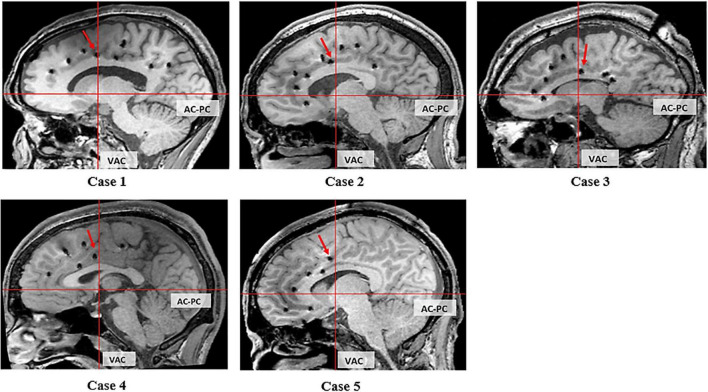
Sagittal T1 MRI of 5 cases. The red arrow indicates the electrode evoking grasping/groping.

Electrical stimulation of the electrode at the ventral bank of the cingulate sulcus in front of the VAC line elicited grasping actions in four patients (Figure 4: cases 1, 2, 4, and 5). In case 3, this location is at the ventral bank of the cingulate sulcus just at the posterior margin of the VAC line (Figure 4). Case 3 had extensive gliosis and hemispheric atrophy from perinatal ischemia distorting the normal anatomy of the brain. Considering this alteration in anatomy, we argue the localization of the grasping action in case 3 may not truly be discordant with the other four patients.

We related the location with the three CMAs buried within the cingulate sulcus (Picard and Strick, 1996, 2001; Amiez and Petrides, 2014). In cases 1, 2, 4, and 5 this corresponds with the RCZp, and in case 3 this corresponds with CCZ.

### Motor Response Other Than Grasping on Electrical Stimulation

We applied the same protocol to examine grasping actions in all the other electrodes implanted in the CMAs buried in the cingulate sulcus and the pMCC. In four other patients (number of electrode contact = 7), we produced other motor phenomena on electrical stimulation of CMAs distinct from the region producing grasping actions. Other motor phenomena included: movement of lips and tongue in one patient from the ventral bank of the cingulate sulcus anterior to the region producing grasping, rhythmic flexion, and extension of the contralateral hand in one patient when stimulating the ventral bank of the cingulate sulcus just anterior to the region producing lip and tongue movement, tonic posturing of the contralateral lower limb in one patient, and tonic posturing of the contralateral upper limb, trunk, and head in one patient when stimulating the ventral bank of the cingulate sulcus posterior to the VAC line. Grasping actions were not found with stimulation in other regions of the cingulate sulcus and pMCC. Locations of all the cingulate electrodes are shown in Figure 2.

## Discussion

This study provides anatomofunctional correlation of the grasping action prospectively in a subject-by-subject manner, using T1 volumetric MRI and normal FDG PET. In our study, grasping actions were produced from functionally intact CMAs in all five cases. The epileptogenic zone did not include the cingulate cortex in any of these patients. All but one case had structurally normal brains on MRI. While case 3 showed hemispheric atrophy, the PET glucose metabolism was normal, suggesting intact cingulate function. Grasping actions were not elicited during stimulation of other implanted regions of the cingulate cortex. Our study demonstrates that grasping actions can be elicited by electrical stimulation of a precise, localized region of the cingulate cortex in the ventral bank of the cingulate sulcus, anterior to the VAC line, as depicted on T1 volumetric MRI (Figure 4). This region corresponds to the CMAav described by Chassagnon et al. (2008).

Electrical stimulation studies reporting grasping are sparse. The majority of these studies detail single-case reports or retrospective reviews of clinical stimulation protocols (Talairach et al., 1973; Chassagnon et al., 2008; Caruana et al., 2018). Talairach et al. (1973) described grasping movements elicited from ACC, in front of the VAC line. This region corresponds with MCC described by Vogt (2009), however, the precise localization of stimulation, relative to the cingulate sulcus, could not be determined as the study was conducted before MRI technology. More recently, Chassagnon et al. (2008) described four patients who displayed grasping mechanisms; one showing an overt grasping behavior during stimulation of the ventral bank of the cingulate sulcus in the cingulate motor area (identified as CMAav), two showing slight gestures of grasping movements when stimulating the CMA at the dorsal bank of the cingulate sulcus in front of vertical AC line (identified as CMAad) and one showing grasping from the pre-SMA at the vicinity of the cingulate sulcus. The localization of grasping in all but one patients is similar to that found in our cases. Caruana et al. (2018) retrospectively analyzed reaching and grasping actions from 35 contacts clustered in the ventral bank of the cingulate sulcus within the aMCC and suggested that this region corresponds with the ventral sector of Brodmann area 24c (Palomero-Gallagher et al., 2009; Amiez and Petrides, 2014). While this study has provided valuable support for the involvement of the cingulate cortex in the grasping mechanism, the electrode positions were based on postoperative MRI. Oane et al. (2020) described integrated gestural automatisms directed to personal or peripersonal space in a retrospective study localized in the aMCC. Their localization is similar to that described by Caruana et al. (2018).

It is interesting to note that we have elicited other motor movements, e.g., tonic posturing and rhythmic movement of tongue/lips/hand, respectively, from posterior and anterior to the region eliciting the grasping action. We did not elicit any motor movements on stimulating the electrodes located in the aMCC. Caruana et al. (2018) described complex motor movement, e.g., getting up impulse, body directed movement, and simple motor movement, e.g., twitches and tremors and negative motor effects elicited from the ventral aMCC, overlapping with the region producing reaching and grasping. Oane et al. (2020) also reported simple elementary motor movement, mostly tonic or clonic contraction and versive movement. They have found localization for the motor movement in the pMCC and PCC, and versive head and eye movement in the pMCC. The localization of tonic posturing in our two patients is similar to that found by Oane et al. (2020).

Both the Caruana et al. (2018) (for the patients after 2009) and Oane et al. (2020) applied post-implantation CT co-registered with pre-implantation MRI brain by using FreeSurfer for localization of the electrodes. Electrodes of interest were subsequently reconstructed on the Fs-LR-average brain template. For patients implanted before 2009, Caruana et al. (2018) used Talairach coordinates and vascular anatomy for the determination of electrode position. The disparity of the localization of grasping between these studies and our data might be explained by different templates (MNI vs. ICBM—152) and fitting methods (FS-LR vs. SPM12/cingulate flat map). A variability in localization produced by using different templates and fitting methods has been well described in the literature (Lancaster et al., 2007). The position of electrodes adopted onto surface reconstruction, template and automated labeling is inherently approximate and therefore, should be used with some level of caution (Oane et al., 2020). Given the cingulate cortex is highly complex and with considerable intersubject variability, subject-by-subject analysis is important for the anatomofunctional correlation (Amiez and Petrides, 2014).

Of the 23 patients who had depth electrodes implanted in the cingulate cortex, only five demonstrated stimulation-mediated grasping actions. This was likely due to the location of contacts on the implanted electrodes, as depicted in Figure 2. Electrical stimulation only elicited a grasping mechanism when stimulating the pMCC, anterior to the VAC. This accords with the localization findings of Chassagnon et al. (2008). Caruana et al. (2018) retrospectively analyzed the effects of stimulation on goal-directed behavior, observing phenomena such as getting-up impulses, reaching and grasping, body-directed actions, and exploratory eye-head movements. There is a growing consensus within the research that goal-directed behaviors require the integration of both sensory and motor information (Caruana et al., 2018; Edwards et al., 2019). According to Edwards et al. (2019), successful sensorimotor integration contributes to the shape and efficiency of motor planning and execution, relative to a given task. While this ability has traditionally been localized to cortical regions, such as the primary motor cortex, posterior parietal cortex, and primary somatosensory cortex (Hatsopoulos and Suminski, 2011), our findings suggest that sensory-evoked movements (i.e., involving sensory input) can be elicited from the cingulate cortex. These findings accord with previous literature which has demonstrated more reliable grasping actions in the presence of sensory input particularly visual stimuli (Chassagnon et al., 2008; Horinouchi et al., 2018).

Research exploring the effects of cingulate stimulation have predominately elicited grasping mechanisms in the absence of tactile sensory input (Talairach et al., 1973; Chassagnon et al., 2008; Caruana et al., 2018). One explanation may be that proprioceptive input compensated for restricted sensory input during stimulation (e.g., increased awareness of one’s body in space in the absence of sensory input). While we observed grasping actions in the absence of sensory input in two cases, grasping was elicited with sensory input in all patients from the same location. This reflex-like phenomenon with electrical stimulation has not been observed in the literature. In the context of goal-directed behavior, sensory integration involves visual, tactile, and proprioceptive input when generating movement (Makino et al., 2016). However, research has found that these sensory abilities can be compensatory, as observed by Chen et al. (2018) who found that proprioceptive cues assisted in accurate grasping when visual input was restricted. Our study seems to support previous research suggesting that the grasping mechanism is a goal-directed behavior that involves sensorimotor integration, with regional involvement of the cingulate motor cortex.

The distinction between whether stimulation evokes a sensory-based or reflex-based grasping mechanism is important in the context of certain neurological disorders, such as stroke. Sensorimotor integration is commonly disrupted following a stroke, particularly as the middle cerebral artery supplies blood to both motor and sensory regions (Walcott et al., 2014). Paresis in the upper extremities, such as the hands, is common after stroke and can have chronic effects on hand movements (Lang et al., 2013). As the integration of sensory and motor information is critical to motor control, an understanding of which regions serve sensorimotor functions is integral when developing potential therapeutic solutions. Our findings regarding the nature of the grasping mechanism following pMCC stimulation may add to the growing body of literature implicating this region in the network physiology of goal-oriented, complex hand movements. Deep brain stimulation may therefore provide an exciting avenue that could be explored in certain neurological disorders that are characterized by disruption of goal-directed movement (e.g., Parkinson’s disease).

Studies utilizing artificial grasping are sparse. One study has utilized surface electrodes over the muscles to produce artificial grasping by electrical stimulation (Ferrari de Castro and Cliquet<suffix>Jr.</suffix>, 2000). Targeting the peripheral system does, however, have several disadvantages, such as requiring multiple surface electrodes to achieve a coordinated grasping and increased risk of infection. Stimulation of artificial grasping may be life-changing for patients with hand paralysis. However, a unique possibility of DBS for this purpose has not been explored, likely due to a lack of understanding of the precise mechanisms and localization of grasping.

### Stimulation Parameters and Future Clinical Application of Grasping

Understanding the safety limits and optimal parameters of electrical stimulation is as important as the precise localization of the grasping action for future research and clinical purposes. We measured the charge density to determine the safety of the current as charge density and charge per phase are essential determinants of tissue injury in animal models (Lang et al., 2013). The relationship between these two factors can be expressed in an equation known as the Shannon equation (Shannon, 1992). The recommended safety limit of charge density as per the Shannon equation is 30 μC/cm^2^ per pulse (Kuncel and Grill, 2004). However, a subsequent study in humans, using subdural grid electrodes, showed a safe charge density of 52–57 μC/cm^2^ per pulse (Gordon et al., 1990). The effect of frequency and duration of the current are not examined (Kuncel and Grill, 2004).

In this study, the charge density required for generating grasping actions was 18–54 μC/cm^2^, except for visual reaction in two patients which required a higher charge density of 72 μC/cm^2^. Although the latter is higher than the recommended safety limit, this is in line with the stimulation parameters applied by Caruana et al. (2018) (frequency = 50 Hz, pulse width = 1 ms, current intensity = 0.4–5 mA, duration of stimulation = 5 s; and calculated charge density = 8–100 μC/cm^2^) (Talairach et al., 1973; Chassagnon et al., 2008; Caruana et al., 2018). Other centers routinely apply charge densities of 54–60 μC/cm^2^ for electrical stimulation during SEEG (Gordon et al., 1990; Shima et al., 1991). One study used high currents of 15–20 mA (50 Hz frequency, 0.3 ms pulse width) with grid and depth electrodes, applying lower current with depth electrodes (Ramasubbu et al., 2018). However, charge density could not have been calculated as the area of the depth electrode in contact with the cortical tissue stimulated was not mentioned by the author.

One key finding of our study was that the latency of onset and duration of the grasping action was influenced by certain stimulation parameters, specifically the intensity of the current. This observation highlights the importance of considering stimulation parameters when recreating localized functions and has crucial clinical implications for stimulation-based therapeutic solutions (e.g., DBS in Parkinson’s disease).

The application of electrical stimulation in movement disorder is well established (Ramasubbu et al., 2018). Early hypotheses regarding the functional mechanism of DBS were based on the observation that electrical stimulation can generate similar effects to a lesion in the same region (e.g., pallidotomy in Parkinson’s disease; Herrington et al., 2016). The charge density applied in DBS ranges from 1.37 to 45.8 μC/cm^2^/phase for the treatment of Parkinson’s disease, essential tremor, dystonia, and obsessive-compulsive disorder (Fakhar et al., 2013). However, the frequency of current applied in DBS is much higher 100–150 Hz (Wang et al., 2009; Lozano et al., 2012). While the charge density required to elicit grasping in our study was higher than DBS, the frequency of current was significantly lower. We think the electrical parameters in the current study can be applied safely for future research and clinical purpose.

Grasping is an essential function of the hand. Taking the analogy of DBS in Parkinson’s disease, brain stimulation may be applied in the region of the cingulate cortex to produce controlled, complex hand movements in patients with hemiplegia, however, devoted research is needed to explore this in the context of stroke patients. Our study sheds some light on the localization of the grasping mechanism and provides insight into the stimulation parameters required to consistently reproduce this movement.

## Data Availability Statement

The data that support the findings of this study are available from the corresponding author upon reasonable request.

## Ethics Statement

The studies involving human participants were reviewed and approved by the WSLHD (Western Sydney Local Health District) Human Research Ethics Committe. The patients/participants provided their written informed consent to participate in this study.

## Author Contributions

ZR was the primary investigator, responsible for the study design, data collection, analysis, and drafting of the manuscript for intellectual content. NM helped with the intellectual contents and drafting the manuscript. AB and VF independently reviewed all the videos and revised the manuscript for intellectual content. MB helped with the data collection. JS-P helped with the data collection and examination of patients during the electrical stimulation. NM independently examined the grasp reflex during the electrical stimulation. CW independently examined the grasp reflex during the electrical examination and revised the manuscript for the intellectual content. MD was responsible for the implantation of the electrodes. All authors contributed to the article and approved the submitted version.

## Conflict of Interest

The authors declare that the research was conducted in the absence of any commercial or financial relationships that could be construed as a potential conflict of interest.

## Publisher’s Note

All claims expressed in this article are solely those of the authors and do not necessarily represent those of their affiliated organizations, or those of the publisher, the editors and the reviewers. Any product that may be evaluated in this article, or claim that may be made by its manufacturer, is not guaranteed or endorsed by the publisher.

## Supplementary Material

Supplementary Video 1 (Index case) Groping (left hand) on electrical stimulation (ES). Note that ipsilateral hand (right side) did not show any groping during ES.

Supplementary Video 2 Strong grasp reaction. Note: GR stopped after cessation of ES.

Supplementary Video 3 Magnet reaction.

Supplementary Video 4 Trap reaction.

Supplementary Video 5 Visual groping which could be aborted with command.

Supplementary Video 6 Closing reaction.
